# The New Biomarker for Cervical Squamous Cell Carcinoma and Endocervical Adenocarcinoma (CESC) Based on Public Database Mining

**DOI:** 10.1155/2020/5478574

**Published:** 2020-04-12

**Authors:** Hao Ding, Xiao-Xing Xiong, Guan-Lan Fan, Yue-Xiong Yi, Yu-Rou Chen, Jing-Tao Wang, Wei Zhang

**Affiliations:** ^1^Department of Gynecology, Zhongnan Hospital of Wuhan University, Wuhan 430071, China; ^2^Central Laboratory, Renmin hospital, Wuhan University, 430006, China

## Abstract

To reconstruct the ceRNA biological network of cervical squamous cell carcinoma and endocervical adenocarcinoma (CESC) and to select an appropriate mRNA as a biomarker that could be used for CESC early diagnosis and prognosis evaluation. We downloaded CESC data from the TCGA public database, and statistical analysis was conducted with the R software to find out differential expressed genes encoding for lncRNAs, miRNAs, and mRNAs. The differentially expressed mRNAs (DEmRNAs) screened in the ceRNA network were analyzed for survival to find the mRNAs with significantly linked to the survival prognosis. These mRNAs were searched in the Pathological Atlas to identify the final appropriate mRNAs. Differential expression analysis revealed 773 lncRNAs, 94 miRNAs, and 2466 mRNAs. Survival analysis of DEmRNAs in the ceRNA network indicated that ADGRF4, ANXA8L1, HCAR3, IRF6, and PDE2A (*P* < 0.05) were negatively correlated with survival time. Verification of these six DEmRNAs in the Pathology Atlas indicated that PDE2A was a possible biomarker for CESC patients. PDE2A might be a biomarker for early diagnosis and prognosis evaluation of CESC patients, but due to the lack of available data, further studies may be needed for confirmation.

## 1. Introduction

Cervical cancers are a leading cause of mortality among women [1], especially in developing countries [2], and they are the second most common gynecological cancer type [3]. At the same time, a large number of patients are diagnosed with cervical cancer every year [4]. Among cervical cancers, the cervical squamous cell carcinoma and endocervical adenocarcinoma (CESC) kind account for 10-15% of all female cancer-related deaths and present the second-highest mortality behind breast cancer [5]. However, until now, medical diagnosis methods have not provided a good biomarker to detect CESC patients early enough. In most cases, patients have already progressed into invasive stages when the cancer is detected. In addition, more issues are appearing. For example, the incidence age is lower [6], and morbidity incidence, as well as recurrence rate, is becoming higher [7]. Therefore, it is important and urgent to find novel biomarkers that can predict the occurrence or evaluate the prognosis of cervical squamous cell carcinoma and endocervical adenocarcinoma (CESC) patients as early as possible.

However, this is not an easy task to do, because the occurrence and development of CESC are a very complex biological process [8], involving molecular, genomics, proteomics, and other biological metabolic processes. Among the participants in these biological processes, the most interesting biomarkers are all the kinds of RNA in cells, including long noncoding RNAs (lncRNAs), microRNAs (miRNAs), and messenger RNAs (mRNAs). In recent years, research scientists worked to discover the biological link among noncoding RNAs and coding RNAs. Approximately 98% of the human genome is transcribed into noncoding RNAs [9], suggesting many unknown effects on physiological and pathological processes. Research indicates that miRNAs can suppress the translation and induce the degradation of mRNAs, modulating gene expression and function [10], so miRNAs play a critical role in tumor genesis, while lncRNAs were shown to participate in many disease [11] processes. However, the functional role of lncRNAs in CESC is still unknown.

Generally speaking, lncRNAs mainly have a function in chromatin regulation, transcriptional regulation, and regulation of alternative splicing in the nucleus [12]. However, lncRNAs also adsorb related miRNA through competitive endogenous RNA (ceRNA) [13] and affect mRNA stability and translational regulation in the cytoplasm. The ceRNA hypothesis was first proposed in 2011 [14]. The ceRNA interaction network includes the three vital elements, lncRNAs, miRNAs, and mRNA. lncRNAs act as an endogenous molecular sponge, competitively binding miRNAs via shared miRNA response elements with reverse complementary binding seed regions, and thus indirectly regulating mRNA expression levels [15]. Many scientific studies have now confirmed the ceRNA hypothesis in hepatocellular carcinoma [16], breast cancer [17], and nonsmall cell lung cancer [18]. However, analyses of the CESC ceRNA network are rare and there is a lack of verification of the corresponding clinical data.

**Figure 1:**
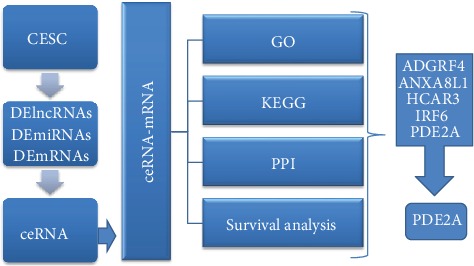
The flowchart of ceRNA network construction of CESC; ceRNA: competitive endogenous RNA network; DElncRNAs: differential lncRNA; DEmiRNAs: differential miRNA; DEmRNAs: differential mRNA; ceRNA-mRNA: mRNAs in the ceRNA network; GO: GO enrichment; KEGG: KEGG PATHWAY enrichment; PPI: protein-protein interactions.

The Cancer Genome Atlas (TCGA) platform is a well-known open-source sequence database, which covers more than 30 human cancer types and contains a large amount of clinical and bioinformatics data [19]. It has been an important research database for researchers all over the world. Using information downloaded from the TCGA platform, we were able to analyze the ceRNA network. This may help to elucidate the specific biological mechanisms underpinning CESC progression and suggest appropriate biomarkers. The overall flowchart is shown in (Figure 1).

## 2. Subjects and Methods

### 2.1. Data Sets and Preprocessing

Transcriptome RNA-sequencing data of count file of lncRNA, miRNA, and mRNA and clinical information of CESC patients were downloaded from the TCGA data platform (https://tcga-data.nci.nih.gov/tcga/). The screening condition was “Project: TCGA-CESC,” “Experimental Strategy: RNA-Seq,” and “Workflow Type: HTSeq-Counts,” containing 306 CESC Primary Tumor tissues and 3 Solid Normal tissues. lncRNA, miRNA, and mRNA transcription profiles and clinical information of CESC are publicly available and open access. Therefore, approval by a local ethics committee was not needed. Annotation information for lncRNA, miRNA, and mRNA was obtained from the human GENCODE project (https://www.gencodegenes.org/).

### 2.2. Identification and Analysis of Differentially Expressed Genes (DEGs)

We used the edgeR package (R version 3.6.1) to normalize and analyze significantly differentially expressed lncRNAs, miRNAs, and mRNAs, the selection criteria being (|log2 fold change| ≥ 2.0 and FDR adjusted *P* less than 0.05) as determined using the Benjamini-Hochberg method [20]. Differentially expressed lncRNAs (DElncRNAs), miRNAs (DEmiRNAs), and mRNAs (DEmRNAs) were represented on volcano plots and heatmaps.

### 2.3. Reconstruction of the ceRNA Network

We used the weighted gene coexpression network (WGCNA) algorithm [21] implemented in the WGCNA R package (R version 3.6.1) to reconstruct the ceRNA network. The network was then visualized using Cytoscape [22] 3.7.2 and its topology analyzed with the network analysis plugin MCODE, which computed the possible communities (dense clusters) in the network [23].

### 2.4. Functional Analysis of mRNAs in the ceRNA Network

DAVID (https://david.ncifcrf.gov/) was used to validate mRNAs in the ceRNA network, to identify enriched pathways in KEGG (Kyoto Encyclopedia of Genes and Genomes, https://www.genome.jp/kegg/) PATHWAYS and biological processes and cell components in Gene Ontology (GO, http://www.geneontology.org/). *P* values <0.05 were used in all enrichment analysis.

### 2.5. Protein-Protein Interactions of Proteins Encoded by mRNAs in the ceRNA Network

The protein-protein interaction network formed by proteins encoded by mRNAs part of the ceRNA network was constructed using the STRING database [24], and the network was visualized using Cytoscape.

### 2.6. Statistical Analysis and Survival Analysis of DEmRNAs

For overall survival analyses, the survival R package (R version 3.6.1) was used to analyze the DEmRNAs in the ceRNA network between CESC and normal samples. A *P* value <0.05 was considered as statistically significant.

### 2.7. Validation of DEmRNAs in the Pathology Atlas

The Human Protein Atlas database [25] (https://www.proteinatlas.org) maps all the human proteins in cells, tissues, and organs. It can help to better understand the expression of different proteins in normal and pathological tissues and provide valuable information to further identify meaningful biological biomarkers.

## 3. Result

### 3.1. Differentially Expressed lncRNA (DElncRNAs), miRNA (DEmiRNAs), and mRNA (DEmRNAs)

**Figure 2:**
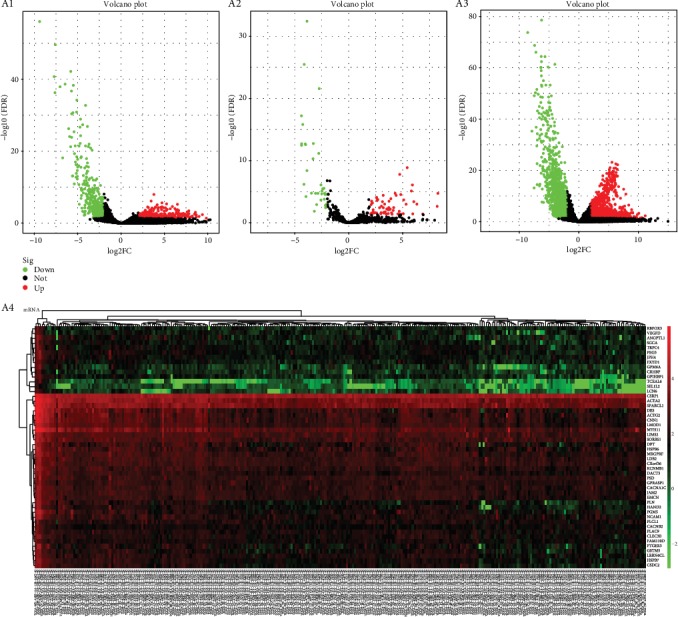
The volcano and heatmap plots of differentially expressed RNAs; A1: differential lncRNA; A2: differential miRNA; A3: differential mRNA; A4: the heatmap plots for differentially expressed mRNAs (DEmRNAs) for top 50 genes. The horizontal axis represents samples from the TCGA database. The vertical axis represents RNAs. Red denotes upregulated genes, and green denotes downregulated genes.

Using 306 CESC Primary Tumor tissues and 3 Solid Normal tissue expression profiles of lncRNAs, miRNAs, and mRNAs, we identified a total of 2466 DEmRNAs (upregulated 1103, downregulated 1363), 773 DElncRNAs (upregulated 331, downregulated 442), and 94 DEmiRNAs (upregulated 60, downregulated 34). Differentially expressed RNA-encoding genes are shown on volcano plots and heatmaps (Figure 2).

### 3.2. Reconstruction and Analysis of lncRNA-miRNA-mRNA ceRNA Network and Identification of Hub Nodes

**Figure 3:**
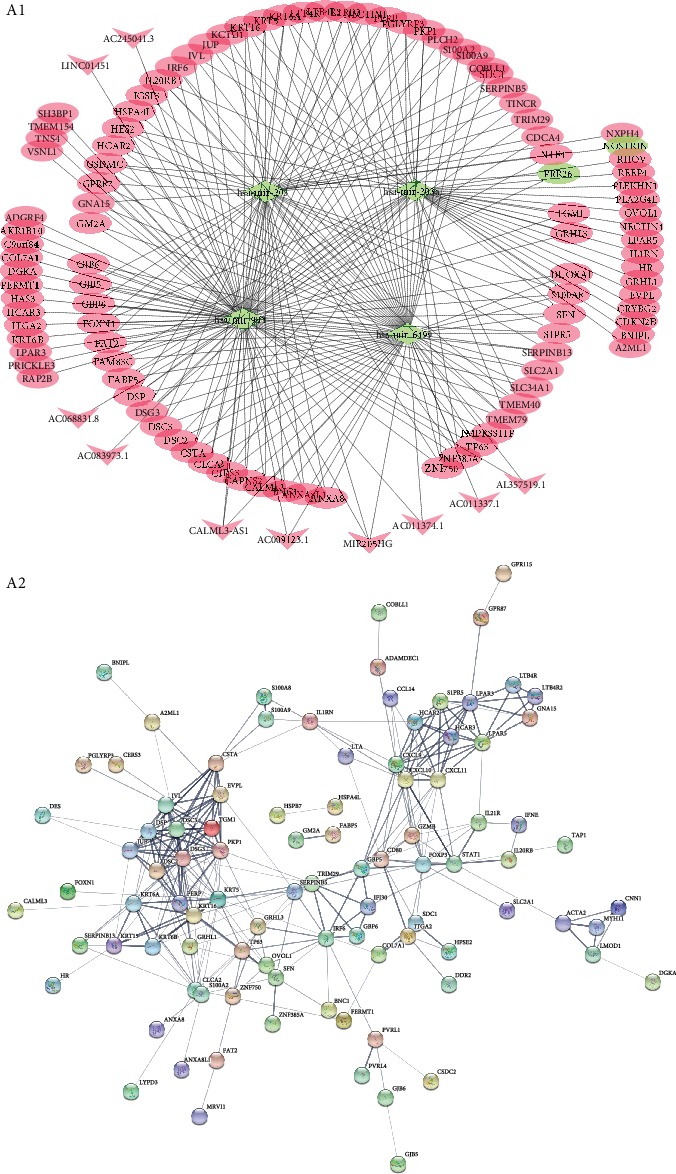
The lncRNA-miRNA-mRNA ceRNA network and protein-protein interaction network; A1: the lncRNA-miRNA-mRNA ceRNA network, the arrow nodes denote lncRNA, the diamond nodes denote miRNA, and the ellipse nodes denote mRNA. The red indicates upregulation and green indicates downregulation; A2: the protein-protein interaction network from the STRING database. Each nodes represent mRNA-encoded proteins. Connections between nodes represent the relationship between proteins.

The lncRNA-miRNA-mRNA ceRNA network was reconstructed based on the relationship between differentially expressed lncRNAs, miRNAs, and mRNAs determined with WGCNA and visualized by Cytoscape, the selected soft threshold power parameter were 5,6,5 in the subnetwork of lncRNA-mRNA, lncRNA-miRNA, and miRNA-mRNA (Supplementary Material ([Supplementary-material supplementary-material-1])). The CESC ceRNA network comprises 156 nodes and 293 edges (Figure 3). hsa-mir-944, hsa-mir-6499, hsa-mir-205, and hsa-mir-203a appear to be hub nodes.

### 3.3. Functional Enrichment Analyses for DEmRNAs

The Gene Ontology (GO) project [26] provides structured, controlled vocabularies, and classifications that cover several domains of molecular and cellular biology, freely available for community use. Geneset GO enrichment performed with DAVID shows that DEmRNAs are mainly enriched in plasma membrane, extracellular, exosome, and extracellular region. KEGG PATHWAY [27] enrichment analysis shows enrichment in cytokine-cytokine receptor interaction, Arrhythmogenic right ventricular cardiomyopathy (ARVC), and Toll-like receptor signaling pathway. These results are shown in (Table 1).

### 3.4. Protein-Protein Interactions of Proteins Encoded by DEmRNAs

The protein-protein interaction network among proteins encoded by DEmRNAs in ceRNA was constructed using the online tool STRING database, and the network was visualized using Cytoscape. The complete network contains 144 nodes and 285 edges, as shown in (Figure 3).

### 3.5. Statistical Analysis and Survival Analysis of DEmRNAs

**Figure 4:**
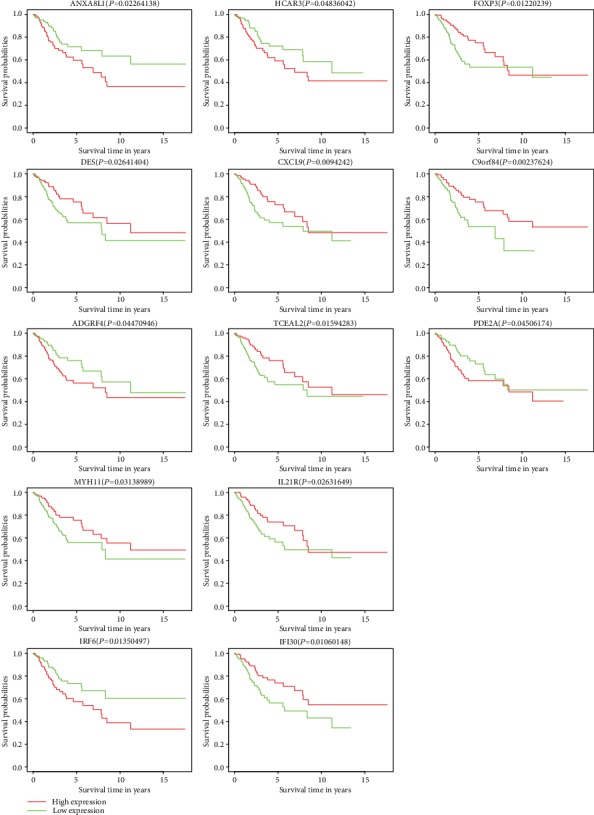
The overall survival curves for differentially expressed mRNAs (DEmRNAs) in the ceRNA network. Horizontal axis: overall survival time; Vertical axis: survival function.

To further identify the DEmRNAs associated with prognosis in 306 ESCC patients, all the DEmRNAs were analyzed with the survival R package Statistical significance at *P* < 0.05. Survival analysis of all the clinical data shows that the expression of 13 DEmRNAs (C9orf84, CXCL9, DES, FOXP3, IFI30, IL21R, MYH11, ADGRF4, ANXA8L1, HCAR3, IRF6, PDE2A, and TCEAL2) was correlated to the prognosis of patients, among which ADGRF4, ANXA8L1, HCAR3, IRF6, and PDE2A were negatively correlated to survival time (Figure 4).

### 3.6. Further Filtering and Validation of DEmRNAs in the Pathology Atlas

**Figure 5:**
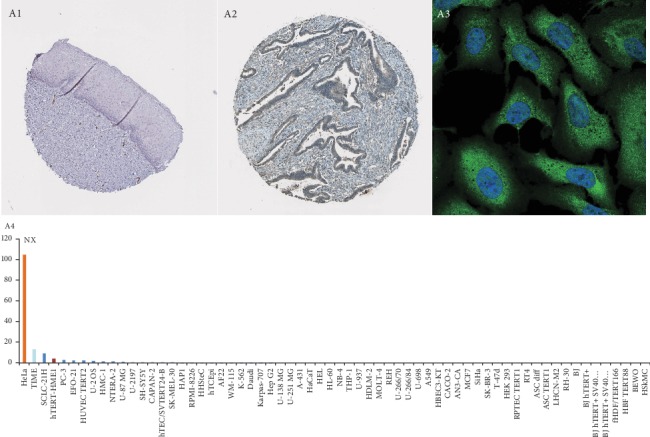
The expression of PDE2A in normal uterine glandular and squamous epithelial tissue and cervical adenocarcinoma tissue from the Human Protein Atlas database; A1: PDE2A show not stained in normal uterine tissue; A2: PDE2A show stained in cervical adenocarcinoma tissue; A3: the green fluorescence show PDE2A detected in cytosol use the U2-OS human osteosarcoma cell line; A4: the PDE2A RNA expression in different cell lines from the Human Protein Atlas database.

The Human Protein Atlas database [25] was used to verify the expression of ADGRF4, ANXA8L1, HCAR3, IRF6, and PDE2A in normal and pathological cervical tissues. Ignoring DEmRNAs for which there was no pathological data, we found that there was a differential expression of PDE2A in normal cervical tissues and pathological cervical tissues. PDE2A also presents a high expression in HeLa cell lines, one of cervical cancer cell lines (Figure 5).

## 4. Discussion

Cervical cancer ranks as the second [28] most common gynecological cancer type and poses a great threat to women's health. In most cases, patients have already progressed into an advanced stage when they are diagnosed. Concurrent chemoradiotherapy plus brachytherapy are the standard treatment options for cervical cancer patients [29]. However, the prognosis of patients is poor. Therefore, researchers are looking to screen out a suitable biomarker for early diagnosis and prognostic assessment. mRNAs encoding proteins in the tumor tissue play an important role in the development, recurrence, progression, and metastasis of cancer cells. In recent years, noncoding RNAs [30] have been an active field of research, attracting the attention of many researchers. Since mRNAs are located at the heart of many biological metabolic processes, selecting the appropriate mRNA as a biomarker is more meaningful. The core of the ceRNA biometabolic network is made up of lncRNAs, which affect mRNA stability and translational regulation in the cytoplasm through competitive endogenous RNA (ceRNA) [31] regulation mechanisms, adsorbing miRNAs. This provides a novel research approach that can enable us to better understand the metabolic relationships between lncRNAs, miRNAs, and mRNAs. We thus hope to narrow our search of biomarkers that have biological relationships with tumor cells to more relevant mRNA.

Downloading CSEC data from the TCGA database, using the weighted gene coexpression network (WGCNA) algorithm [21] implemented in the WGCNA R package to reconstruct the ceRNA network, and by survival analysis of the corresponding clinical data, we found that the expressions of a total of 13 DEmRNAs (C9orf84, CXCL9, DES, FOXP3, IFI30, IL21R, MYH11, ADGRF4, ANXA8L1, HCAR3, IRF6, PDE2A, and TCEAL2) were correlated to the prognosis of patients, among which ADGRF4, ANXA8L1, HCAR3, IRF6, and PDE2A were negatively correlated to survival time. Then, by searching the Human Protein Atlas, ignoring the mRNAs for which there is pathological data, PDE2A mRNA was identified as a potential biomarker, with low expression in normal cervical tissue and high expression in tumoral cervical tissue.

PDE2A is highly and specifically expressed in the brain and in certain tumors and involved in the pathophysiology of related diseases [32]. PDE2A is a member of the Phosphodiesterase family (PDE), a family of enzymes that metabolically inactivate the second messengers (cAMP and cGMP) through hydrolysis of the cyclic phosphate and are therefore critical for the termination of the corresponding signaling cascades [33]. Phosphodiesterase 2A (PDE2A) is a dual substrate enzyme hydrolyzing both cAMP and cGMP, both play vital roles as intracellular second messengers [34]. Existing research shows that PDE2A is mainly expressed in brain tissue [35], especially highly expressed in the cortex, amygdala, and hippocampus, but with relatively little expression detected in the midbrain, hindbrain, and cerebellum. Peripheral tissues also display low levels of expression [33]. Among other human tissues, PDE2A is mainly expressed in adrenal glands and vascular endothelial cells, and it has been speculated that PDE2A may be related to vascular permeability [36]. Through Watanabe et al.'s [37] research, we know that PDE2A can regulate the expression of miR-139, and miR-139 was significantly associated with lymph node metastasis and histological invasiveness in nonsmall cell lung cancer (NSCLC). A study on adrenocortical carcinoma (ACC) [38] showed that miR-139-5p, which gene is located in the 11q13.4 locus within intron 2 of PDE2A, was overexpressed in ACC patients with poor prognosis. Overexpression of PDE2A was associated with vascular invasion in colorectal cancer cell lines [39], finds overexpressed PDE2A associated with vascular invasion in colorectal cancer cell lines. Doecke et al.'s [40] research proved that PDE2A was significantly associated with survival time in kidney renal papillary cell carcinoma. In early research, professor Hiroshi et al. [41] showed PDE2A expression in malignant melanoma PMP cells. Shen et al. [39] have also shown that PDE2A expression was upregulated in colorectal cancer. Finally, He et al. [42] have shown that PDE2A was significantly associated with the prognosis of liver cancer, using ceRNA network analysis and Multivariate Cox regression analysis.

Most of the current researches on PDE2A are pursued in neuroscience. Conversely, there is very little literature on the direct relationship between PDE2A and tumors, and its involvement in the mechanism of appearance and development of tumors is not clear. Even in neuroscience, the research on PDE2A is still in its infancy. Some researchers [43] have proved that PDE2A inhibitors have the potential for the treatment of cognitive disorders. Gomez et al. [33] have shown that PDE2A inhibitors are useful for the treatment of memory disorders while Nakashima et al. [44] demonstrated that PDE2A inhibitors improve cognitive impairments in rat models of schizophrenia. Other studies [45] have shown that PDE2A is involved in regulating signal transduction in lung macrophages, and upregulated PDE2A [46] expression contributed to lung injury in mice models [47]. Altogether, these early studies have shown that we lack PDE2A research data, and that research focuses on neuroscience. Due to the lack of detailed clinical and experimental data, we cannot accurately evaluate the potential of PDE2A as a biomarker for CESC. However, taking into consideration the expression of PDE2A in pathological tissues from the Human Protein Atlas database, it might be a good biomarker for CESC patients. This provides an encouraging direction for our subsequent research, in order to further illustrate and understand PDE2A expression in human normal and tumoral tissues. Validating whether PDE2A is suitable as a biological indicator in early diagnosis and evaluation of the prognosis of CESC patients is very important.

## 5. Conclusions

Based on the analysis of data from public databases, PDE2A might be a biomarker for early diagnosis and prognosis evaluation of CESC patients, although the lack of detailed clinical and experimental data requires further studies.

## Figures and Tables

**Table 1:** Functional analysis for mRNAs in the ceRNA network.

Category	Term	Count	*P* value
KEGG_PATHWAY	hsa04060: Cytokine-cytokine receptor interaction	8	4.92E-03
KEGG_PATHWAY	hsa05412: Arrhythmogenic right ventricular cardiomyopathy	5	2.66E-03
KEGG_PATHWAY	hsa04620: Toll-like receptor signaling pathway	5	1.34E-02
Category	Term	Count	*P* value
GOTERM_CC_DIRECT	GO:0005886~plasma membrane	51	1.54E-04
GOTERM_CC_DIRECT	GO:0070062~extracellular exosome	41	3.03E-05
GOTERM_CC_DIRECT	GO:0005576~extracellular region	20	3.25E-02
GOTERM_CC_DIRECT	GO:0005615~extracellular space	19	1.22E-02
GOTERM_BP_DIRECT	GO:0008544~epidermis development	15	4.45E-15
GOTERM_MF_DIRECT	GO:0005509~calcium ion binding	15	1.17E-03
GOTERM_MF_DIRECT	GO:0042802~identical protein binding	14	4.82E-03
GOTERM_BP_DIRECT	GO:0007155~cell adhesion	12	1.07E-03
GOTERM_BP_DIRECT	GO:0006954~inflammatory response	11	8.78E-04
GOTERM_BP_DIRECT	GO:0006955~immune response	11	1.92E-03
GOTERM_BP_DIRECT	GO:0030216~keratinocyte differentiation	10	8.50E-09
GOTERM_MF_DIRECT	GO:0005198~structural molecule activity	10	1.19E-04
GOTERM_CC_DIRECT	GO:0005913~cell-cell adherens junction	10	7.61E-04
GOTERM_BP_DIRECT	GO:0006915~apoptotic process	10	3.65E-02
GOTERM_CC_DIRECT	GO:0005856~cytoskeleton	9	7.04E-03
GOTERM_CC_DIRECT	GO:0030054~cell junction	9	2.28E-02

## Data Availability

The lncRNA, miRNA, and mRNA expression datasets and clinical information of CESC patients were downloaded from the TCGA data platform (https://tcga-data.nci.nih.gov/tcga/).

## Supplementary Materials

Supplementary Materials Supplementary Figure 1: the related soft threshold power parameter about the subnetwork to construct the ceRNA. (a) The subnetwork of lncRNA-mRNA. (b) The subnetwork of lncRNA-miRNA. (c) The subnetwork of miRNA-mRNA.
