# Supplementary figures and images for: The New Biomarker for Cervical Squamous Cell Carcinoma and Endocervical Adenocarcinoma (CESC) Based on Public Database Mining
Source: Biomed Res Int. 2020 Apr 12;2020:5478574. doi: 10.1155/2020/5478574 (PMC7174939; doi:10.1155/2020/5478574)

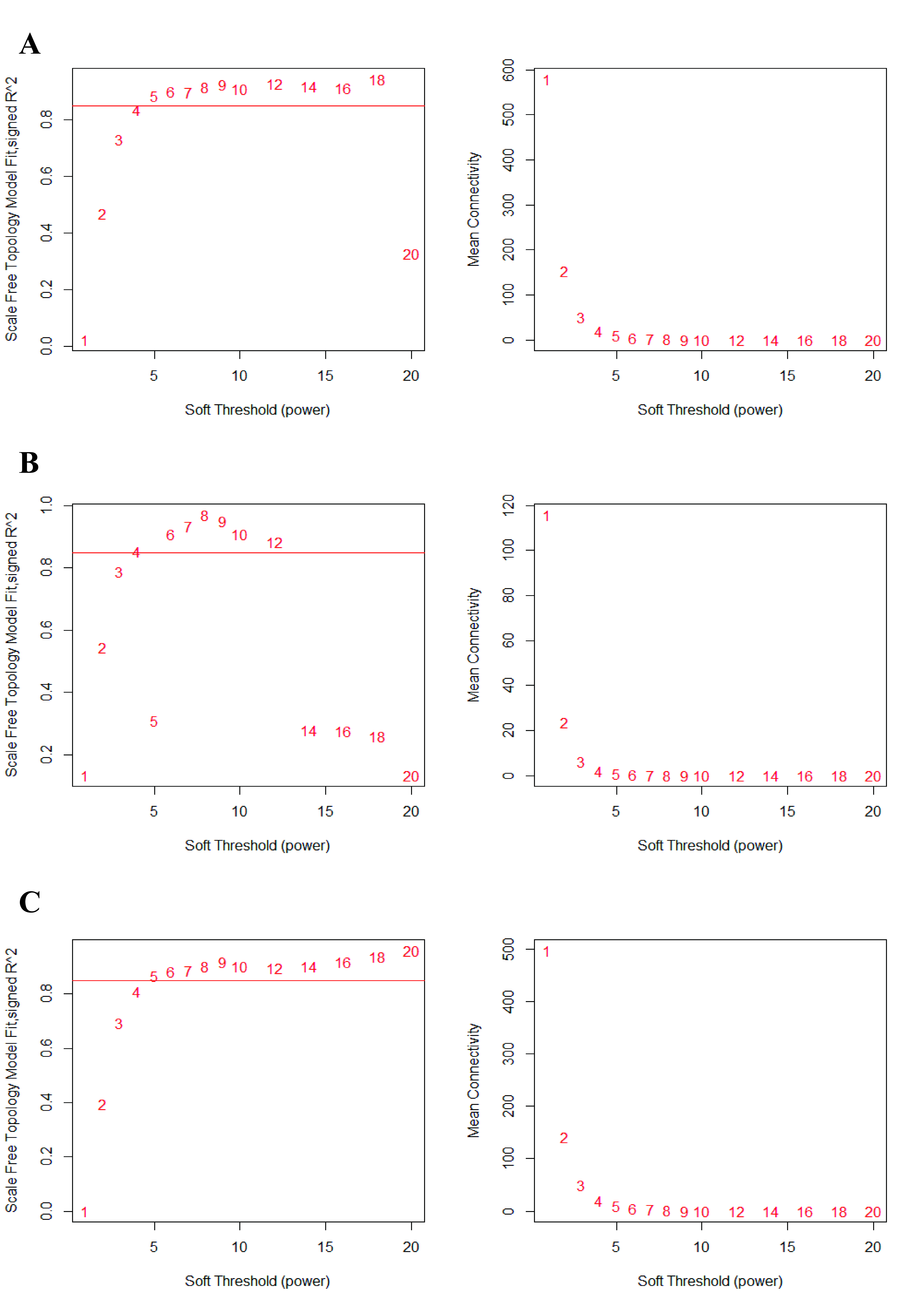

Supplement: Supplementary Materials — Supplementary Figure 1: the related soft threshold power parameter about the subnetwork to construct the ceRNA. (a) The subnetwork of lncRNA-mRNA. (b) The subnetwork of lncRNA-miRNA. (c) The subnetwork of miRNA-mRNA. [file 5478574.f1.jpg]
